# N-Propyl-N-Methylpyrrolidinium Difluoro(oxalato)borate as a Novel Electrolyte for High-Voltage Supercapacitor

**DOI:** 10.3389/fchem.2019.00664

**Published:** 2019-10-09

**Authors:** Weili Zhang, Fuming Zhang, Peng Zhang, Shuo Liang, Zhiqiang Shi

**Affiliations:** Tianjin Key Laboratory of Advanced Fibers and Energy Storage, College of Materials Science and Engineering, Tianjin Polytechnic University, Tianjin, China

**Keywords:** N-propyl-N-methylpyrrolidinium difluoro(oxalato)borate, supercapacitors, ionic liquid, electrolyte, high voltage

## Abstract

Development of high voltage electrolyte is one of the effective ways to improve the performance of supercapacitor. The new ionic liquid N-propyl-N-methylpyrrolidinium difluoro(oxalato)borate (Py_13_DFOB) was designed and mixed with propylene carbonate (PC) as electrolyte for supercapacitor. The operating voltage of the new electrolyte system has been proven to be up to 3.0 V by a series of electrochemical techniques. Surprisingly, the new salt exhibits nearly symmetric capacitance contribution in the positive and negative electrodes, leading to a high capacitance value of 130 F g^−1^. The energy and power density of EDLCs using Py_13_DFOB in the PC electrolyte reach 39.06 Wh kg^−1^ (100 mA g^−1^) and 8.03 kW kg^−1^ (5,000 mA g^−1^), respectively, at the working voltage of 3.0 V, significantly exceeding the performance of commercial electrolyte tetraethylammonium tetrafluoroborate (TEABF_4_). The results indicate that Py_13_DFOB can be a promising electrolyte salt for supercapacitor.

## Introduction

Supercapacitors (SCs), a kind of electrochemical energy storage device with high power density, long cycle life, and excellent reliability, has been widely used in many fields such as hybrid electric vehicle and high-power output equipment. However, the low energy density is the main factor hindering their further applications (Wang et al., 2012; Simon et al., 2014). It can be obtained from the calculation formula of energy density that expanding the voltage window of the cell is the most effective way for achieving high energy supercapacitor (Li et al., 2007; Snook et al., 2011; Boukhalfa et al., 2012; Díaz et al., 2012; Kato et al., 2012; Okashy et al., 2013; Xiang et al., 2013; Borenstein et al., 2014, 2015; Choi et al., 2014; Kumar et al., 2016). As a matter of fact, the working voltage V depends to a great extent on the stability of the electrolyte. Unfortunately, tetraethylammonium tetrafluoroborate (TEABF_4_) as the state-of-the-art electrolyte material can only withstand a working voltage of 2.5–2.7 V, which is usually limited by the oxidation and reduction stability of electrolyte ions.

Recently, Ionic liquids (ILs) have been intensively studied and viewed as potentially ideal electrolytes for increasing the operating voltage of EDLCs due to their relatively wide electrochemical stability (Ue et al., 2003; Zhu et al., 2007; Kim et al., 2014). In addition, ILs are attracted to supercapacitors owing to several other excellent properties in terms of non-volatile, non-flammable, and high thermal stability. However, most ILs are trapped in their low ionic conductivity and high viscosity compared to aqueous electrolytes and even organic electrolytes. Considering this situation, mixing ILs with organic solvents is a promising alternative strategy to enlarge the working voltage without sacrificing the power density and cycle life of the EDLCs (Guerfi et al., 2010; Kühnel et al., 2011). The introduction of organic solvent not only reduces the viscosity and increases the conductivity of the pure ionic liquid, but also maintains a large electrochemical stability window, which greatly improves the capacitive performance of the device.

The most conventional families of ionic liquids, which have been evaluated as the most prospective electrolytes for supercapacitors, are based on pyrrolidinium and imidazolium cations (Mousavi et al., 2016; Watanabe et al., 2017). In general, pyrrolidinium based ILs could deliver noticeably enhanced electrochemical stability than the ones based on imidazolium owing to its superior ability to resist oxidation and reduction, which are suitable for realizing novel symmetric supercapacitor with high working voltage (Lin et al., 2011; Brandt et al., 2013; Zhang et al., 2016; Martins et al., 2018). Moreover, the mixing of the ionic liquid with the organic solvent can substantially ignore the high viscosity of the pyrrolidinium-based ionic liquid. The choice of anions also has a great effect on the properties of the ionic liquid. Difluoro(oxalate)borate (DFOB) have received particular interest in recent years due to its high asymmetry compared to conventional anions, resulting in higher solubility of electrolyte salt in ester solvents. Also, the electron-withdrawing fluorine atom on DFOB leads to more delocalization charges, causing lower affinity of binding cation and higher conductivity of electrolyte salt. More importantly, DFOB possesses high electrochemical stability and non-corrosive to aluminum current collector, rendering it an ideal choice for high-voltage electrolytes (Lai et al., 2011; Allen et al., 2013; Tian et al., 2014; Wu et al., 2015). In this manuscript, we designed a new ionic liquid Py_13_DFOB mixed with an organic solvent PC as electrolyte for supercapacitor. The physicochemical properties (conductivity, melting point, thermal stability, etc.) of the new electrolyte salt were characterized for the first time. Afterwards, we evaluated the performance of supercapacitors containing Py_13_DFOB/PC electrolyte from the aspects of withstand voltage, cycle stability, energy density, power density, etc.

## Experimental

### Materials

N-Methyl pyrrole (>99%), 1-Bromopropane (>98%), acetonitrile (>99.5%) were obtained from Aldrich and used without further purification. The PC solvent (battery grade, extra dry <20 ppm of water) and Lithium difluoro(oxalato)borate (>99%) were purchased from Jiangsu Guotai Super Power New Materials Co. Ltd. (China). The prepared electrolyte salt (Py_13_DFOB) was placed in a glove box filled with high pure argon (<1 ppm O_2_ and <1 ppm H_2_O), then dissolved in the PC with 1 mol L^−1^ concentration, added 3Å molecular sieves to remove trace moisture for 1 week. The final water content is <20 ppm testing by Karl Fischer titration method (Mettler-Toledo C20, Switzerland). The impurities of halide and alkali metal ions were <2 ppm confirmed by Inductive Coupled Plasma Emission Spectrometer (ICP) test.

The activated carbon electrodes were prepared by mixing 82% activated carbon (Kuraray YP-50), 10% carbon black (VXC72) as the conductor, 4% carboxymethylcellulose sodium (CMC), and 4% styrene butadiene rubber (SBR) as the binder. The mixture was stirred to a sticky state followed by coating on aluminum foils. The electrodes were punched into disks with a diameter of 18 mm, then dried under vacuum at 120°C for more than 12 h prior to be used. The mass of each electrode is about 6 mg and the thickness is ~60 um (including 20 um aluminum foil).

### Electrolyte Synthesis/Purification

N-Methyl pyrrole (8.11 g, 0.1 mol) and Lithium difluoro(oxalato)borate (14.38 g, 0.1 mol) were dissolved in 100 mL acetonitrile. To this solution, 1-Bromopropane (12.99 g, 0.1 mol) was added dropwise for 12 h. The crude products were obtained by filtration and rotary evaporation of the reaction solution and then purified by extraction with ethyl acetate/deionized water. Finally, the target product of high purity clear liquid was obtained after decoloring by activated carbon. The resulting electrolyte salts are dried for 48 h at 80°C and stored in a sealed container in a glove box with high pure argon (<1 ppm O_2_ and <1 ppm H_2_O).

### Characterization and Measurements

^1^H-NMR and ^13^C-NMR spectra were performed on a Bruker AVANCE 400M spectrometer. Thermal gravimetric analysis was tested by a Netzsch thermogravimetric analyzer at a heating rate of 5°C min^−1^ from 20 to 600°C under nitrogen conditions. Differential scanning calorimetry (DSC) analysis was performed on a TA Instruments Q2000 differential scanning calorimeter at a heating rate of 10°C min^−1^ under nitrogen atmosphere. The relationship between conductivity and concentration of Py_13_DFOB/PC was determined by using a conductivity meter (Mettler-Toledo S30, Switzerland). The viscosity of the electrolyte is tested by a viscosity testing device (A&D SV-10, Japan). Activated Carbon/Activated carbon symmetrical coin cells were prepared in an argon-filled glove box for electrochemical measurements of electrolytes. The galvanostatic charge/discharge tests (GCD) and cycling performance were tested at Arbin battery test system. Cyclic voltammetry (CV) was tested in the same range by Autolab electrochemical workstation (PGSTAT302N, Switzerland). The gravity specific capacitance of single electrode was obtained from equation C_m_ = 2*I*Δt/*m*Δ*V*, in which *I* is the current value, Δt is the discharge time, Δ*V* is the potential difference between the end of the voltage drop and the end of the discharge, and *m* is the mass of the active material of single electrode. The energy density is calculated from the formula E = 1/2*C*Δ*V*^2^, where *C* is the specific discharge capacitance. The power density was calculated by the equation *P* = *I*Δ*V*/2*m*.

## Results and Discussions

### Chemical Structure and Physical Properties Characterization of Synthesized Sample

^1^H NMR and ^13^C NMR analysis were used to verify the purity of our synthesized sample. The results are as follows: ^1^H NMR (D_2_O) δ: 3.42~3.34 (m,4H), 3.17~3.13 (m,2H), 2.90 (s,3H), 2.12~2.06 (m,4H), 1.74~1.64 (m,2H), 0.87~0.83 (t,3H), ^13^C-NMR (D_2_O): δ = 9.99, 16.75, 21.24, 47.97, 64.17, 65.71, 163.76 ppm, which confirm that Py_13_-DFOB is synthesized successfully. The original NMR spectrum is shown in Figure S1.

**Figure 1:**
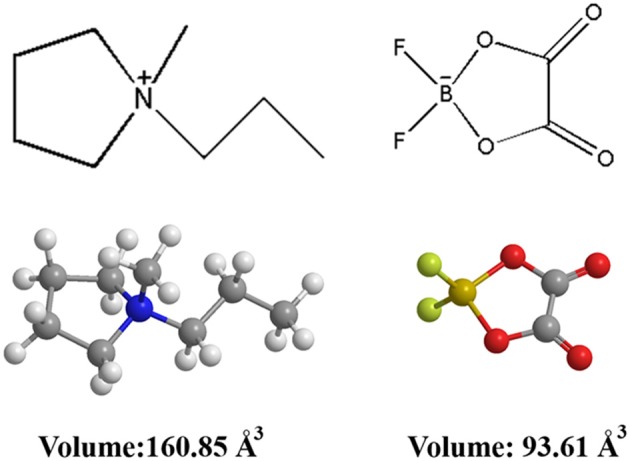
Molecular structures and volume of the electrolyte components.

**Figure 2:**
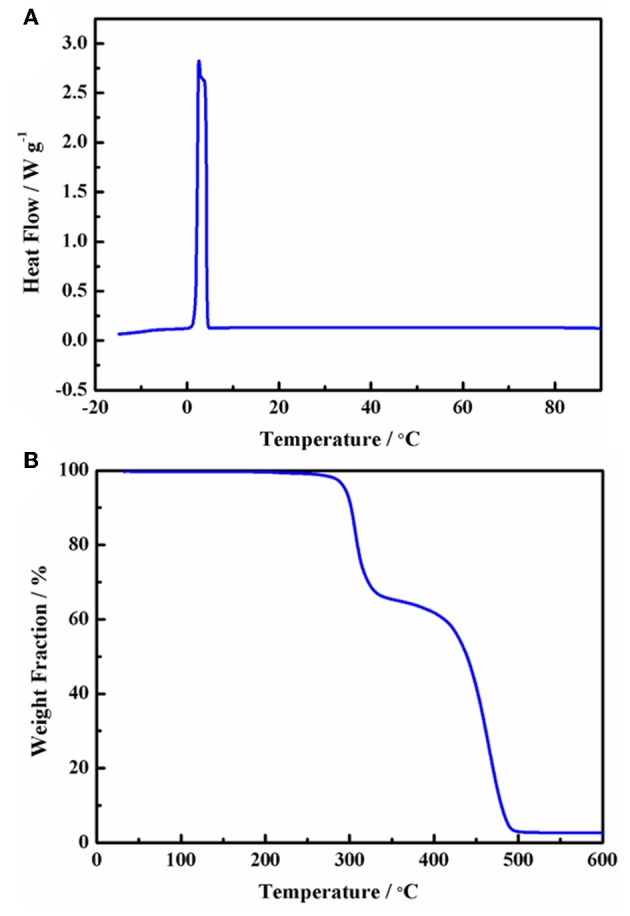
DSC **(A)** and TGA **(B)** heating traces (5°C min^−1^) of the Py_13_DFOB.

The chemical structure and ionic size of the synthesized Py_13_DFOB are shown in Figure 1 and several basic physical properties (conductivity, viscosity, thermal stability, etc.) of the new electrolyte were summarized in Table 1. The phase transformation behavior of Py_13_DFOB investigated by DSC is illustrated in Figure 2A. Py_13_DFOB shows a low melting point value of 3.21°C, which is due to the unfavorable packing of ions and the decrease of lattice energy of IL materials caused by the highly spatial asymmetry of ${\text{Py}}_{13}^{+}$ and the large Vander Waals volume of DFOB^−^. TG analysis is used to observe the thermal stability of electrolytes. As can be seen in Figure 2B, Py_13_DFOB is subjected to two-step degradation, where the initial degradation temperature of 290°C is considered to meet the thermal stability requirements of supercapacitors. The result is similar with the decomposition curve of the LiDFOB mentioned in the previous study (Allen et al., 2011).

**Table 1:** Physical characteristics of Py_13_DFOB.

**Electrolyte**	**Molecular weight (g/mol)**	**Viscosity (mPas)**	**Conductivity (mS cm^**−1**^)**	**Melting point (°C)**	**thermal-decomposition temperature (°C)**
Py_13_DFOB	263.81	105	2.2	3.21	306

**Figure 3:**
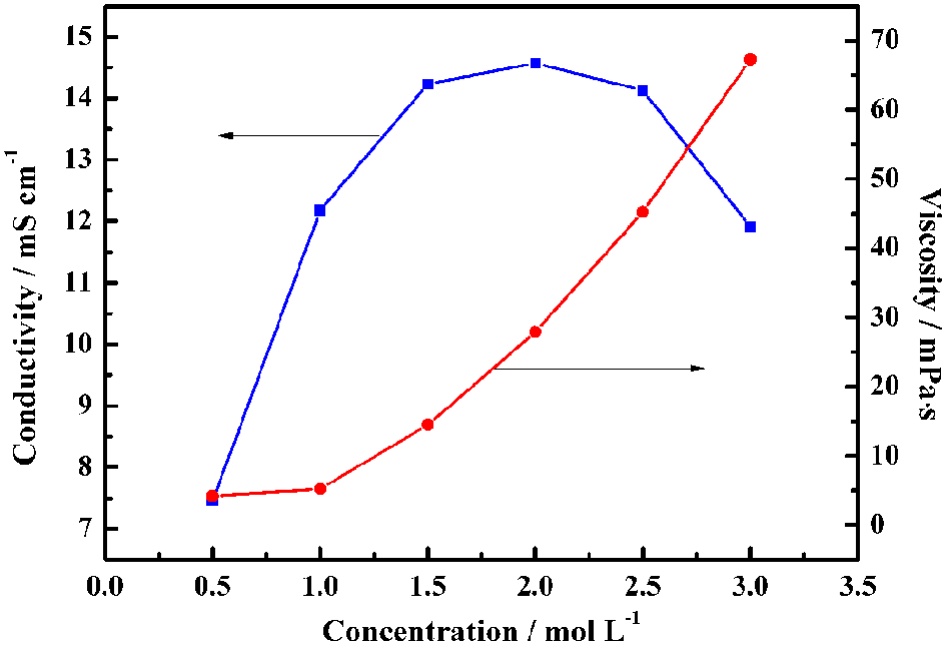
Relationship between conductivity (viscosity) and concentration of Py_13_DFOB/PC at 20°C.

Obviously, Table 1 shows that the pure ionic liquid exhibits lower conductivity and higher viscosity than the organic electrolyte. Therefore, the mobility of the ions is enhanced by the addition of the organic solvent PC, thereby making it easier for the electrolyte to access the pores of the activated carbon. As shown in Figure 3, the maximum conductivity of Py_13_DFOB/PC was observed to reach 14.5 mS cm^−1^ at 2.0 mol L^−1^, and the conductivity drops sharply as the concentration exceeds or <2.0 mol L^−1^. Besides, the addition of the solvent significantly reduces the viscosity of the pure ionic liquid, taking a 1 mol L^−1^ Py_13_DFOB/PC as an example, the viscosity value of which is only 3.8% of the pure ionic liquid. It should be emphasized that 1 mol L^−1^ Py_13_DFOB/PC was selected as the most preferred one to be applied later in the study based on the data in supporting documentation. Therefore, the electrochemical performance of the electrolyte applied to supercapacitors is not only determined by the conductivity, but viscosity is also a factor that cannot be ignored.

### Potential Window Opening: Stability of the Electrolytes

**Figure 4:**
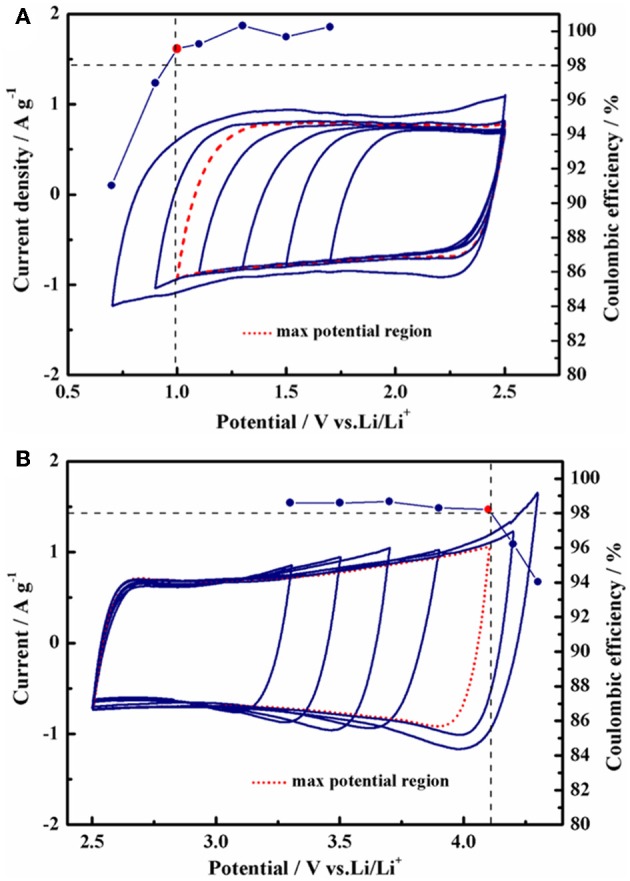
Operating potential determination using cyclic voltammetry. Coulombic efficiency (right axis) of cells used in the determination of anodic/cathodic limits. **(A)** Negative, **(B)** positive scans in different windows of the half-cell using 1 mol L^−1^ Py_13_DFOB/PC.

Compared with TEABF_4_/PC, the supercapacitor using 1 mol L^−1^ Py_13_DFOB/PC displayed higher energy density and power density under conventional working voltage (2.7 V) while maintaining excellent rate performance and long cycle stability (Figure S3). Further, the high voltage characteristics of the new electrolyte system need to be clarified. Since the electrochemical window cannot directly reflect the working voltage of the full cell due to the difference in the structure of anions and cations, it is necessary to identify the real positive and negative stability limits of the new electrolyte in activated carbon electrodes (Fic et al., 2012). For this purpose, we initially evaluated the maximum operating voltage possible of 1 mol L^−1^ Py_13_DFOB/PC via cyclic voltammetry investigations on three electrode cells with an AC-based working electrode, a largely oversized AC-based counter electrode with the same composition and a Li wire reference electrode. An efficiency threshold value of 98% was chosen, and further efficiency declines were considered to be a series of irreversible reactions involving electrolytes in the cell system. The obtained single cell electrochemical window limits are presented in Figure 4. With this condition, the positive and negative potential limits occur at +1.6 V vs. Li and −1.5 V vs. Li, respectively, resulting in a maximum operating voltage of 3.1 V.

**Figure 5:**
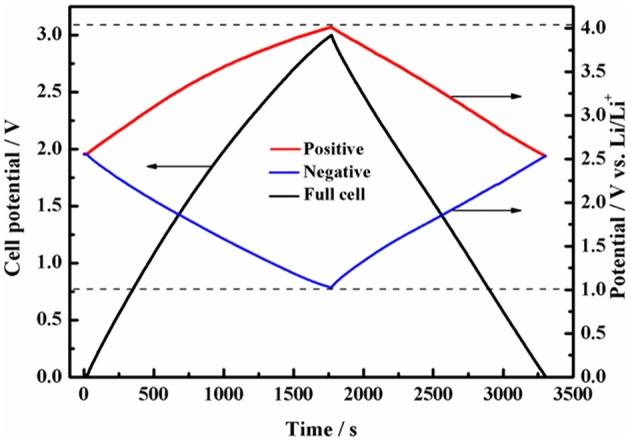
Potential profiles of the positive and negative electrodes during galvanostatic charge/discharge of symmetric supercapacitor with 1 mol L^−1^ Py_13_DFOB/PC.

We further measured the GCD curve of a symmetric full-cell at an operation voltage of 3.0 V while monitoring the capacitive behavior of positive and negative electrodes. It can be seen from Figure 5 that the GCD curves exhibits superior capacitive behavior, and the nearly symmetric potential window of positive and negative electrodes shows that the capacitance contributions of DFOB^−^ anion and ${\text{Py}}_{13}^{+}$ cation are almost the same. More importantly, the cutoff potential of the positive and negative electrodes vs. Li is within the safe potential range when the operating voltage of the full cell reaches 3.0 V.

In previous studies, mass balancing is currently the preferred method to make full use of the maximum working voltage of electrolyte, but it will have a negative impact on the specific capacitance of the full cell due to the extra increase in the mass of one of the electrodes (Weingarth et al., 2013; Van Aken et al., 2015; Hu et al., 2016). Besides, due to the change of ion transfer channel and electronic response time of high-quality electrode, resulting in inconsistent polarity of the two electrodes, thus affecting the cycle life of the cell. Excitingly, the Py_13_DFOB/PC can almost fully exploit its maximum working potential window without requiring additional mass balance, eliminating the time consumption and technical problems of electrode matching, and harvesting higher energy density.

### Electrochemical Study in EDLCs Configuration

**Figure 6:**
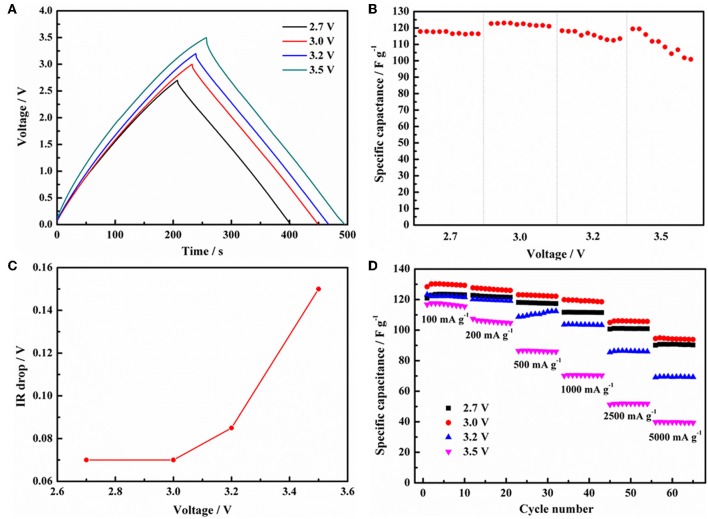
**(A)** GCD curves of the EDLCs with 1 mol L^−1^ Py_13_DFOB/PC at current density of 500 mA g^−1^ with different voltage ranges. **(B)** Specific capacitance and **(C)** IR drop vs. voltage obtained from **(A)** charge-discharge curves. **(D)** The charge/discharge rates performance of EDLCs with 1.0 mol L^−1^ Py_13_DFOB/PC under different voltage ranges.

To evaluate the withstand voltage of EDLCs configuration, the GCD curves of EDLCs based on 1 mol L^−1^ Py_13_DFOB/PC operating at 500 mA g^−1^ under several applied voltages are shown in Figure 6A. The GCD curves maintain a typical triangular shape, and the variation of potential with time shows an approximate linear relationship as the voltage rising to 3.0 V, which demonstrates excellent electrochemical reversibility and stability of EDLCs. However, the charging curve shifts rather than overlaps, and the linearity and symmetry of the GCD curves gradually deteriorated when the voltage exceeds 3.0 V, indicating detrimental processes may be occurring between electrodes and electrolytes. We obtained the same result from the relationship between specific capacitance (Figure 6B), IR drop (Figure 6C) with voltage obtained from the GCD test in Figure 6A, that is, the abrupt change of the curve all occurs when the voltage exceeds 3.0 V. The IR drop is closely related to the equivalent series resistance of the cell, reflecting the state of the working environment inside the EDLCs. When the voltage is raised from 3.0 to 3.5 V, the IR drop abruptly increases from 0.074 to 0.152 V, indicating some irreversible changes in the internal environment of the cell. The rate behavior under different voltages (2.7, 3.0, 3.2, 3.5 V) was given by the GCD test with a current density ranging from 100 to 10,000 mA g^−1^. As shown in Figure 6D, the discharge capacitance of the cell decreases with increasing current density due to the rise in internal resistance (IR drop) caused by the kinetic limitation at the electrolyte/electrode interface. However, the cell with Py_13_DFOB/PC still shows the most superior capacitance performance and rate performance at the working voltage of 3.0 V. In general, it is proved that the new electrolyte system can work stably at 3.0 V by electrochemical evaluation of single electrode and assembled supercapacitor.

**Figure 7:**
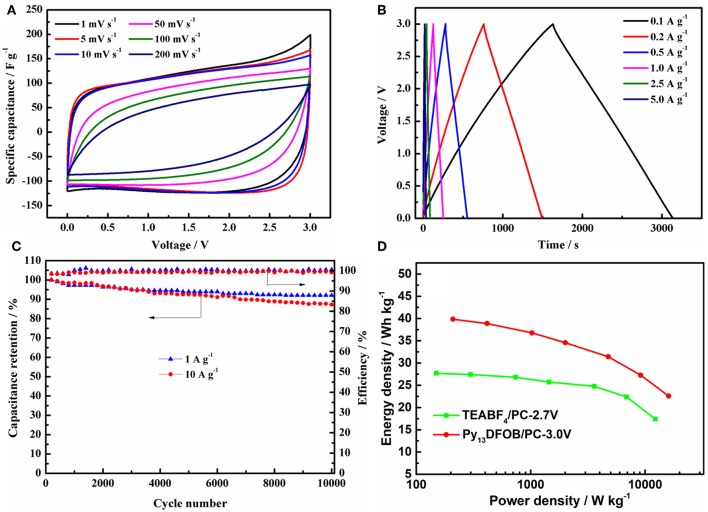
**(A)** GCD curves at different current densities **(B)** CV curves at different scan rates **(C)** cycle performance at current density of 1 A g^−1^ and 10 A g^−1^ under 3.0 V of the EDLCs using 1.0 mol L^−1^ Py_13_DFOB/PC. **(D)** Ragone plots of the EDLCs with two electrolytes.

Next, we evaluated the electrochemical performance of EDLCs containing Py_13_DFOB/PC at 3.0 V operating voltage. Figure 7A showed the CV curves of the cell at different scan rates. The rectangular shape of the CV curves is deteriorated with the increase of the scan rate, but a good rectangular shape is still maintained at the high scan rate, demonstrating the outstanding transfer characteristic of the electrolyte ion. Similarly, GCD curves (Figure 7B) exhibit good linearity, symmetry, and negligible instantaneous voltage drop at different current densities, indicating superior double-layer characteristics. The specific capacitance obtained from the discharge time of the GCD curves can reach a high value of 130 F/g at a current density of 100 mA g^−1^ and still maintain 96 F g^−1^ at 5,000 mA g^−1^ under a working voltage of 3.0 V, which was about 78% of its initial capacitance.

With the aim to determine whether Py_13_DFOB/PC can operate steadily for a long time under the working voltage of 3.0 V, the charge-discharge performance of 10,000 cycles were recorded at current density of 1 and 10 A g^−1^. Figure 7C conveys a clear message that Py_13_DFOB/PC presents excellent long cycle performance at different current densities, delivering capacity retention of 92% at 1 A g^−1^ and 87% at 10 A g^−1^ after 10,000 cycles with nearly 100% coulombic efficiency, confirming the long-term electrochemical stability of Py_13_DFOB /PC at high working voltage.

Ragone plots (energy density vs. power density) has been widely used to evaluate the overall performance of a supercapacitor device. From the results summarized in Figure 7D, Py_13_DFOB/PC obtained a considerably higher energy density and power density than TEABF_4_/PC at each current density due to the simultaneous increase in voltage and specific capacitance. It should be noted that the maximum energy density and power density of the supercapacitor based on Py_13_DFOB /PC can reach 39.06 Wh kg^−1^ (100 mA g^−1^) and 8.03 kW kg^−1^ (5,000 mA g^−1^), respectively, when the voltage goes up to 3.0 V.

## Conclusion

In this work, we successfully synthesized a novel ionic liquid Py_13_DFOB as electrolyte salt for supercapacitor. The 1 mol L^−1^ solution formed by the mixture of Py_13_-DFOB and PC shows the closely transport properties as the commercial electrolyte (TEABF_4_/PC), which solves the problems of high viscosity and low conductivity of pure ionic liquids. More importantly, it is proved that Py_13_DFOB/PC can exhibit outstanding capacitance behavior at high operating voltage of 3 V confirmed by several electrochemical testing techniques. Moreover, we found that the nearly symmetric capacity contributions of positive and negative electrodes convey a high specific capacitance value of 130 F g^−1^. The energy density and power density of supercapacitor with Py_13_DFOB can reach 39.06 Wh kg^−1^ (100 mA g^−1^) and 8.03 kW kg^−1^ (5,000 mA g^−1^), respectively. Based on these results, the new electrolyte system is considered to be a promising electrolyte for high-voltage supercapacitor.

## Data Availability Statement

All datasets generated for this study are included in the manuscript/Supplementary Files.

## Author Contributions

All authors listed have made a substantial, direct and intellectual contribution to the work, and approved it for publication.

### Conflict of Interest

The authors declare that the research was conducted in the absence of any commercial or financial relationships that could be construed as a potential conflict of interest.
