# Supplementary material for: N-Propyl-N-Methylpyrrolidinium Difluoro(oxalato)borate as a Novel Electrolyte for High-Voltage Supercapacitor
Source: Front Chem. 2019 Oct 9;7:664. doi: 10.3389/fchem.2019.00664 (PMC6794414; doi:10.3389/fchem.2019.00664)
Supplement: Supplementary file 1 [file Data_Sheet_1.docx]

Scheme 1. Synthesis route of target compounds


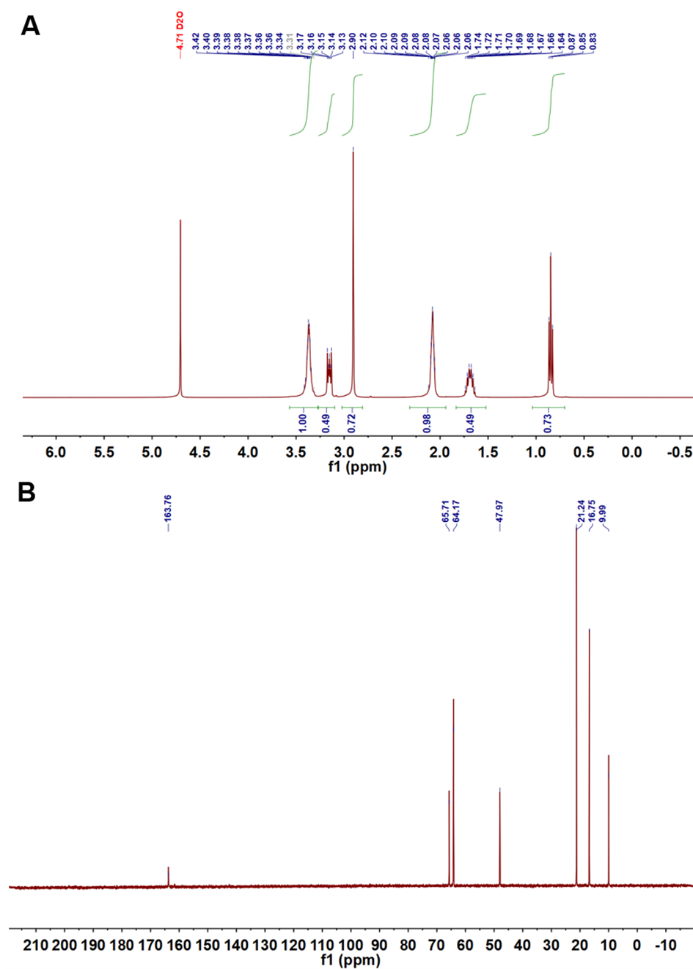


Figure. S1 The ^1^H (A) and ^13^C (B) NMR spectra of the synthesized sample


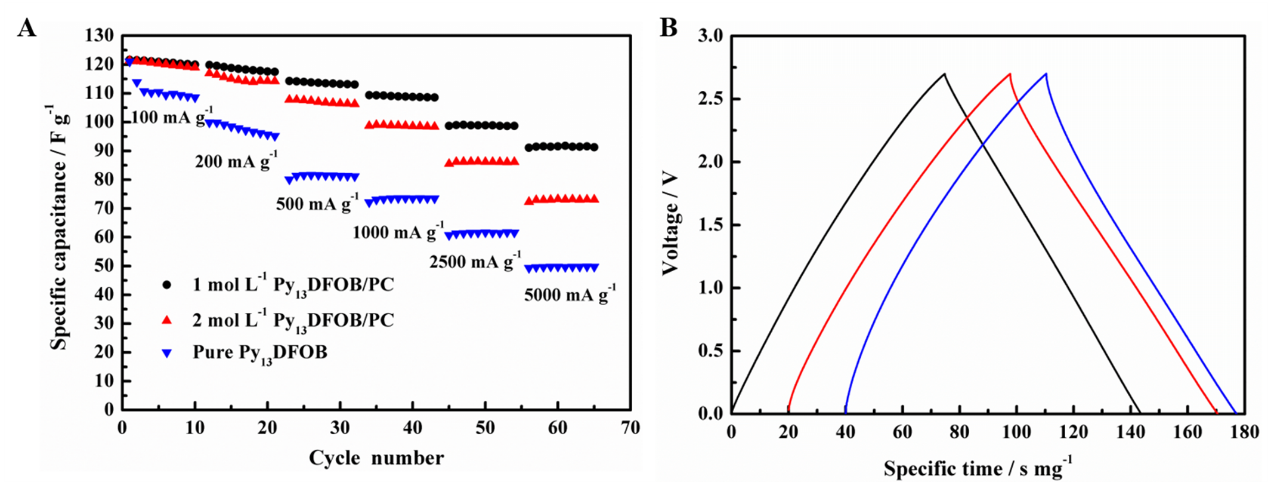


Figure. S2 (A) rate performance of supercapacitors using the three electrolytes (1 mol L^-1^ Py_13_DFOB/PC, 2 mol L^-1^ Py_13_DFOB/PC and pure Py_13_DFOB) working at 2.7 V. Current density from 100 mA g^-1^ to 5000 mA g^-1^ (B) GCD curves of the three electrolytes at 100 mA g^-1^

In order to obtain the best cell performance, the charge-discharge performance of supercapacitors using the three electrolytes (1 mol L^-1^ Py_13_DFOB/PC, 2 mol L^-1^ Py_13_DFOB/PC and pure Py_13_DFOB) was compared when the new electrolyte system was applied to supercapacitors. As shown in Figure. S2(A), it is not surprising that the pure Py_13_DFOB exhibits the worst rate performance, which is related to the high viscosity and low conductivity of ionic liquids. 1 mol L^-1^ Py_13_DFOB/PC shows the most outstanding rate performance among the three electrolytes, the specific capacitance is 92 F g^-1^ at a current density of 5000 mA g^-1^, corresponding to 76.7 % retention of the capacitance at 100 mA g^-1^. The same results are obtained from the GCD curves of Figure. S2(B). 1 mol L^-1^ Py_13_DFOB/PC shows good electrochemical reversibility and typical double layer characteristics, while the GCD curves of the other two electrolytes deviated significantly from linearity. Based on the above results, 1mol L^-1^ Py_13_DFOB/PC is considered to be the most suitable choice for supercapacitor to greatly compensate for the sluggish ion diffusion of pure ionic liquids.


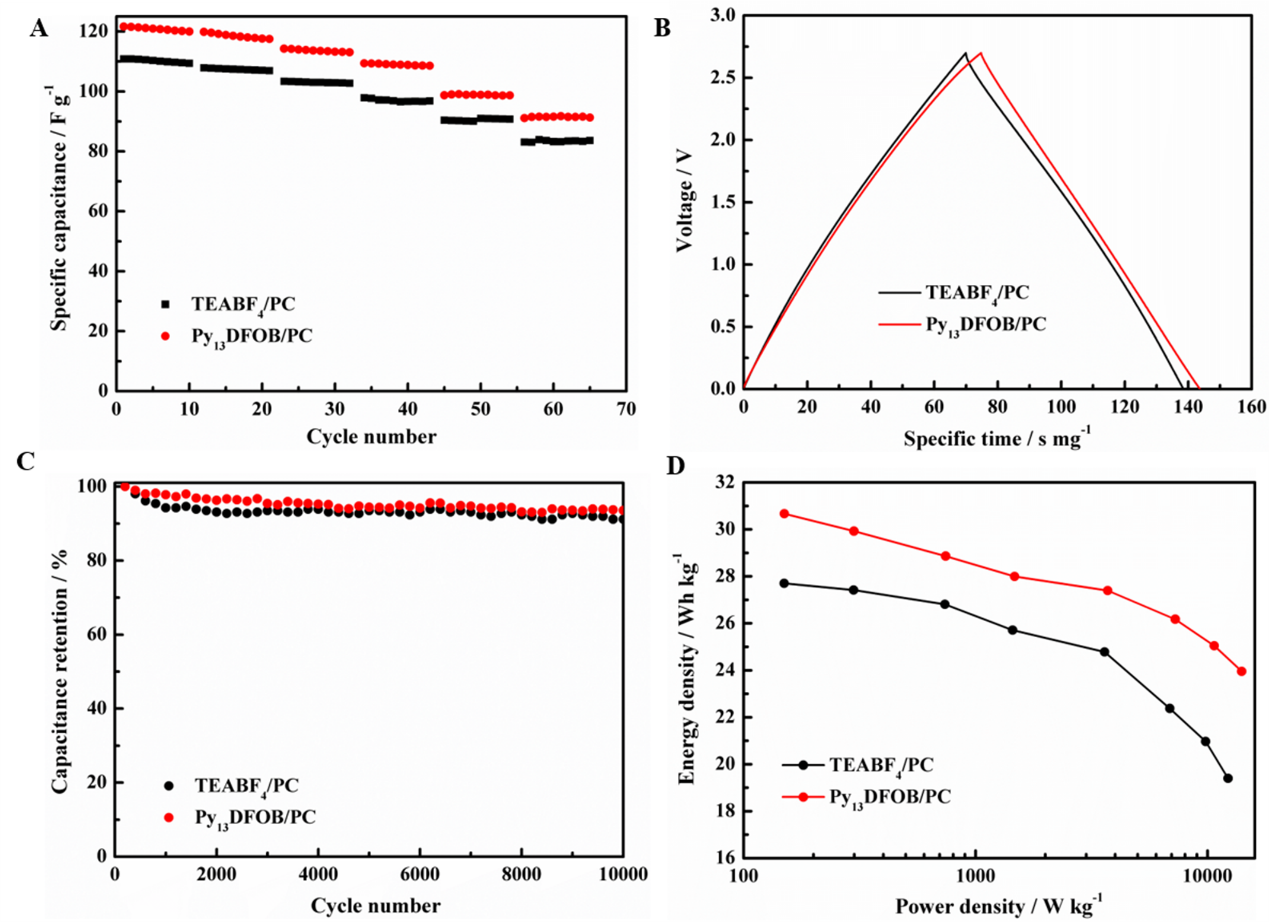
Figure. S3 (A) rate performance of the EDLCs using 1 mol L^-1^ Py_13_DFOB/PC and 1 mol L^-1^ TEABF_4_/PC working at 2.7 V. Current density from 100 mA g^-1^ to 5000 mA g^-1^ (B) GCD curves of the two electrolytes at 100 mA g^-1^ (C) Long cycle performance at current density of 1000 mA g^-1^ under 2.7 V of the EDLCs using the two electrolytes. (D) Ragone plots of the EDLCs with two electrolytes.

At the conventional operating voltage of 2.7 V, the electrochemical performance of EDLCs with 1 mol L^-1^ Py_13_DFOB/PC and 1 mol L^-1^ TEABF_4_/PC were compared. As shown in Figure S3 (A), the rate performance of 1 mol L^-1^ Py_13_DFOB/PC is comparable to that of a commercial electrolyte, but with significantly improved capacitance. Moreover, the GCD curves (Figure S3 (B)) of both electrolytes show good linearity and symmetry, corresponding to the typical dual-electric layer properties. Besides, the capacity retention of EDLCs with both electrolytes after 10,000 cycles is maintained above 90%, demonstrating superior long cycle stability. The EDLCs with 1 mol L^-1^ Py_13_DFOB/PC also gained higher energy density and power density due to higher specific capacitance.
